# The Associations Between Retirement and Cardiovascular Disease Risk Factors in China: A 20-Year Prospective Study

**DOI:** 10.1093/aje/kww166

**Published:** 2017-04-05

**Authors:** Baowen Xue, Jenny Head, Anne McMunn

**Keywords:** cardiovascular disease, China, longitudinal studies, piecewise regression, retirement, risk factors

## Abstract

Despite China's being the largest and most rapidly aging country in the world, there have been no longitudinal studies investigating the relationship between retirement and cardiovascular disease risk factors in China. In this study, we assessed the associations between retirement and systolic blood pressure, diastolic blood pressure, waist circumference, body mass index, smoking status, and alcohol consumption over a 17-year period both before and after retirement among 1,084 people (41.3% women) who participated in the China Health and Nutrition Survey (1991–2011) at least once prior to the year in which they retired and at least once afterward. Piecewise models centered at the year of retirement were applied. Retirement was accompanied by a reduction in diastolic blood pressure, a slowdown in the increase of both systolic blood pressure and waist circumference, and a reduction in the probability of being a heavy alcohol drinker. The association between retirement and blood pressure was stronger for men and for urban dwellers. No significant associations with body mass index or smoking were found. This study suggests that retirement may be beneficial for blood pressure, waist circumference, and alcohol consumption in the Chinese context. Understanding the potential health influence of retirement is essential, given plans to raise the retirement age in China.

Population aging and proposals for delaying retirement in many countries have led to greater interest in understanding the potential long-term associations between retirement and health (1–3). As an important life transition, retirement may influence health by changing financial resources, psychosocial stressors, health behaviors, social integration, and personal control (4, 5).

In terms of health outcomes, cardiovascular disease (CVD) remains the biggest cause of death worldwide (6). When studying the association between retirement and CVD or its risk factors, it is important to consider reverse causality, as poor health may be a determinant of retirement (7), especially for early retirement (8). Longitudinal studies in which CVD risk factors have been measured repeatedly both before and after retirement provide an opportunity to take account of this problem. Using this method, one French study found that coronary heart disease and stroke were not influenced by retirement (9), and an American study showed that postretirement body mass index (BMI) did not change in white-collar employees but increased in blue-collar workers (10). Other longitudinal studies, carried out mostly in Europe and the United States using different methods, have found retirement to be associated with increased CVD (1, 3, 11) and metabolic risk (1, 2, 11), have found no association with chronic conditions (12), or have found associations with increased weight and waist circumference (WC) in blue-collar workers (13, 14).

In China, rates of death from CVD have been increasing (15, 16), and annual numbers of CVD events are predicted to increase by 50% between 2010 and 2030 (17). In addition, population aging is occurring more rapidly in China than in many developed countries (18), so China plans to raise its mandatory retirement age for the first time. The age of mandatory retirement in China is currently 60 years for men, 50 years for blue-collar women, and 55 years for white-collar women. An understanding of the associations between retirement and CVD and CVD risk factors is particularly relevant to China. However, to our knowledge, there is no longitudinal study in China which has examined this. In this study, we aimed to understand the longitudinal association between retirement and objective measures of CVD factors, as well as self-reports of behavioral risk factors, in the China Health and Nutrition Survey (CHNS). We also investigated whether the associations between retirement and CVD risk factors vary by sex or between urban and rural areas.

## METHODS

### Data

The CHNS has collected data in 1989, 1991, 1993, 1997, 2000, 2004, 2006, 2009, and 2011 from randomly selected households in 9 provinces from northern to southern China which vary substantially in terms of economic development, public resources, and health indicators. One of the 9 provinces was added from 1997 onwards. Two cities and 4 counties were selected in each province. Thirty-two urban communities and 30 suburban communities within cities and 32 townships and 96 rural villages within counties were randomly selected in 1989. Twenty households per community/township/village were randomly selected. All individuals in each household were interviewed. Replenishment samples have been recruited since 1997 (19, 20). Data from the 1989 wave were not used in this study, as only preschoolers and adults aged 20–45 years had health outcomes measured in 1989. Survey proposals and the process for obtaining informed consent for the CHNS were approved by the institutional review committees of the University of North Carolina at Chapel Hill, the Chinese National Institute of Nutrition and Food Safety, and the China Center for Disease Control and Prevention. Written informed consent was obtained from each participant.

### CVD risk factors

This study assessed 6 CVD risk factors: systolic blood pressure (SBP), diastolic blood pressure (DBP), BMI, WC, current smoking status, and alcohol consumption. Blood pressure (mm Hg) was measured 3 times after 10 minutes’ seated rest. The mean of the last 2 measurements was used. BMI was calculated as weight (kg) divided by height squared (m^2^). WC (cm) was measured at the point midway between the iliac crest and the costal margin. Current smoking status and number of cigarettes smoked per day were combined, and participants were categorized as nonsmokers, current light/moderate smokers (<20 cigarettes/day), or current heavy smokers (≥20 cigarettes/day) (21). Participants were asked how much alcohol they had consumed each week, on average, during the last year, separately for beer, wine, and liquor. A total amount of pure ethanol consumed per week was calculated assuming the following alcohol content by volume (v/v): 4% for beer, 15% for wine, and 53% for liquor (22). Because the distribution of alcohol consumption data was skewed, consumption was categorized into 3 groups: nondrinker (77%), light/moderate drinker (<60 g/day for men, <40 g/day for women), or heavy drinker (≥60 g/day for men, ≥40 g/day for women) (23).

### Statistical analysis

Among 22,861 adult CHNS participants, we identified 1,121 people who moved from an employment status of “working” to “retired” between 1991 and 2011. In other words, they had participated in the survey at least once prior to the time of retirement and once afterward. Thirty-seven people who retired before age 45 years were further excluded. Therefore, the analysis sample included 1,084 people (636 men and 448 women) with 5,921 observations. Data on WC and alcohol consumption were collected from 1993 onwards; when modeling these 2 outcomes, we only included 970 individuals who retired after 1993.

Piecewise regression is a method of regression analysis in which the independent variable is partitioned into intervals and a separate line segment is fitted to each interval. In this study, we applied piecewise linear regression for continuous outcomes and piecewise logistic regression for categorical outcomes in Stata (StataCorp LP, College Station, Texas), with 2 splines separated at the year of retirement. By testing the differences in the slopes of the regression lines before and after retirement, we were able to evaluate whether risk factors were associated with retirement. This method assumes that the regression lines can change directions but not intercepts at the joint point. Quadratic terms for the 2 splines were included in the piecewise model, if Wald tests showed a better fit with them. For each risk factor, we reported coefficients for slope before retirement and for slope change postretirement.

In every CHNS wave from 1997 onwards, participants who reported that they were not currently working because they were retired were asked about their year of retirement. The first answer given after retirement was used to reduce recall bias. The midyear point between the working wave and the retired wave was used for 49 individuals who did not report their year of retirement and for 36 individuals who only participated in 1991 and 1993. Being retired for less than a year was counted as having been retired for 1 year. Thus, the 20-year follow up from 1991 to 2011 allowed for up to 19 years of observation both before (years −19 to −1) and after (years +1 to +19) retirement. Because of the small sample sizes in the first and last 2 years of observation, we only analyzed years −17 to +17.

To take account of the clustering of the data, analyses using mixed models with repeated measures, individuals, and households as the 3 random-effects levels were conducted in Stata. For continuous outcomes, the models also allowed for random coefficients at the individual level.

### Covariates

Sex, highest educational qualification, and province were included as covariates. Other time-fixed covariates based on the last response before retirement were spouse's working status/no spouse in the household, occupational skill level (using the International Standard Classification of Occupations) (24), physical activity level at the workplace, and per capita annual household income in yuan (using already imputed household income divided by family size and then log-transformed). Smoking status, alcohol consumption, and BMI were also included as time-fixed covariates based on the preretirement time point, except when they themselves were the key outcomes of regressions. Time-varying community urbanization index (constructed by the CHNS) was included as well. When modeling blood pressure as an outcome variable, an indicator of whether participants were taking antihypertensive medication was included as a time-varying covariate. All of the analyses were centered at the year of retirement, so we included age at retirement to adjust for the age effects at each time point. We also included age in 1991 to control for the possibility of period effects that might be influential for particular age cohorts.

### Missing data handling

The percentage of missing data was 6.9% for SBP and DBP, 7.6% for BMI, 6.6% for WC, 4.7% for smoking status, and 5.5% for alcohol consumption. The percentage of missing data for covariates ranged from 0% to 5.3%, with the most missing data being found for whether participants were currently taking antihypertensive medication. Missing outcome and covariate data in the 1,084 participants were multiply imputed in R software (R Foundation for Statistical Computing, Vienna, Austria) using multivariate imputation by chained equations. We did not impute missing data if analytical samples were not present at that wave, and we did not impute missing values for alcohol consumption and WC in 1991, since we only included individuals who retired after 1993 when modeling these 2 outcomes. The imputation procedure included covariates, outcomes, independent variables, and moderators which were used in analysis models. Potential mediators (time-varying household income, fat intake, and energy intake) were also included. Missing values of time-fixed preretirement covariates (BMI, smoking, household income) were not included in the imputation and were constructed from imputed time-varying values.

Imputation of multilevel data is still an open area of research. R software includes the mice.impute.2L.pan() function, which was used to impute missing continuous data under a linear 2-level model. The imputation was performed in a wide format, and it used each individual's identification number as the “class variable” to specify that repeated measures belonged to the same person. Higher levels of the data structure were not incorporated into the imputation. The proportion of total missing values was 23.5%, and 25 data sets were imputed.

### Potential moderators

We assessed whether the associations between retirement and CVD risk factors depended on sex or urbanicity (living in an urban area or a rural area). Rural villages were classed as rural areas, and the remaining areas were classed as urban areas.

## RESULTS

Table 1 shows the descriptive characteristics of the sample. Because many women look after the home full-time or work in informal sectors of the economy, they made up only 41% of the analytical samples. Men retired on average 5.8 years later than women and were socially advantaged in terms of education and occupation, but they were much more likely to be smokers and alcohol drinkers. Half of the adults in the CHNS were from urban areas, but 84% of the selected sample lived in urban areas. This is probably because retirement in our sample was concentrated among urban dwellers, while rural dwellers in China are often unable to take true retirement (25). Compared with rural retirees, urban retirees retired on average 4.2 years earlier, were more socially advantaged in every respect, and were less likely to be smokers or drinkers.

**Table 1.** Preretirement Characteristics of Participants by Sex and Urbanicity in the China Health and Nutrition Survey, 1991–2011

Characteristic	Sex				Urbanicity				Total (*n* = 1,084)	
	Male (*n* = 636)		Female (*n* = 448)		Urban (*n* = 902)^a^		Rural (*n* = 182)^b^			
	Mean (SD)	%	Mean (SD)	%	Mean (SD)	%	Mean (SD)	%	Mean (SD)	%
Retirement age, years	59.5 (6.3)		53.7 (6.3)		56.4 (6.5)		60.6 (7.8)		57.1 (6.9)	
Age in 1991, years	49.2 (8.4)		42.6 (8.7)		45.7 (8.8)		50.1 (9.6)		46.4 (9.1)	
Log household income, yuan	8.6 (1.5)		8.8 (1.7)		8.8 (1.5)		8.2 (1.5)		8.7 (1.5)	
Educational attainment										
No schooling completed		13.7		16.4		12.9		24.3		14.8
Primary school completed		19.2		13.3		15.7		22.1		16.7
Middle school completed		26.1		26.3		25.7		28.7		26.2
High school diploma		15.3		18.4		18.0		9.4		16.6
Technical degree		12.0		16.6		14.1		12.7		13.9
College degree/higher		13.7		9.0		13.6		2.8		11.8
Spouse's employment status										
Working spouse		42.3		71.5		52.9		61.8		54.3
Nonworking spouse		52.5		18.3		40.2		29.2		38.4
No spouse		5.2		10.2		6.9		9.0		7.3
Occupational skill level										
1 (lowest)		14.4		7.3		5.6		40.8		11.5
2		33.3		49.8		42.5		27.9		40.1
3		19.3		24.1		22.1		17.3		21.3
4 (highest)		33.0		18.8		29.8		14.0		27.1
Workplace physical activity										
Very light		36.1		35.2		40.1		13.5		35.7
Light		29.8		39.8		34.3		32.2		34.0
Moderate		18.7		18.3		19.1		15.8		18.5
Heavy/very heavy		15.4		6.7		6.5		38.5		11.8
Smoking status^c^										
Nonsmoker		43.1		97.3		69.3		50.6		66.3
Light/moderate smoker		27.1		2.1		15.9		18.5		16.3
Heavy smoker		29.8		0.6		14.8		30.9		17.4
Alcohol consumption^d^										
Nondrinker		41.8		88.4		63.9		54.4		62.3
Light/moderate drinker		47.7		10.1		30.6		34.4		31.2
Heavy drinker		10.5		1.5		5.5		11.2		6.5

Abbreviation: SD, standard deviation.

^a^ Urban areas included urban communities, suburban communities, and townships. Forty-six percent of urban residents were women.

^b^ Twenty percent of rural residents were women.

^c^ Light/moderate smoking was defined as <20 cigarettes/day; heavy smoking was defined as ≥20 cigarettes/day.

^d^ Light/moderate drinking was defined as <60 g/day for men and <40 g/day for women; heavy drinking was defined as ≥60 g/day for men and ≥40 g/day for women.

### Retirement and CVD risk factors

**Figure 1.**
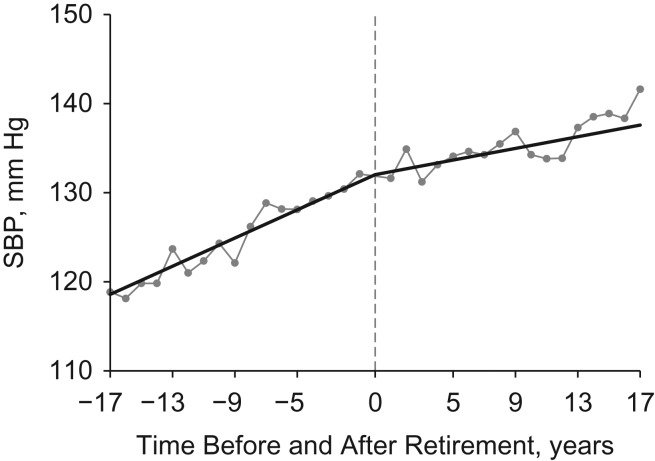
Trajectories of systolic blood pressure (SBP) before and after retirement in the China Health and Nutrition Survey, 1991–2011. The straight lines are the predicted trajectories, separated at the year of retirement. The connected dots are the predicted mean values for SBP in each year. The time of retirement is year 0.

**Figure 2.**
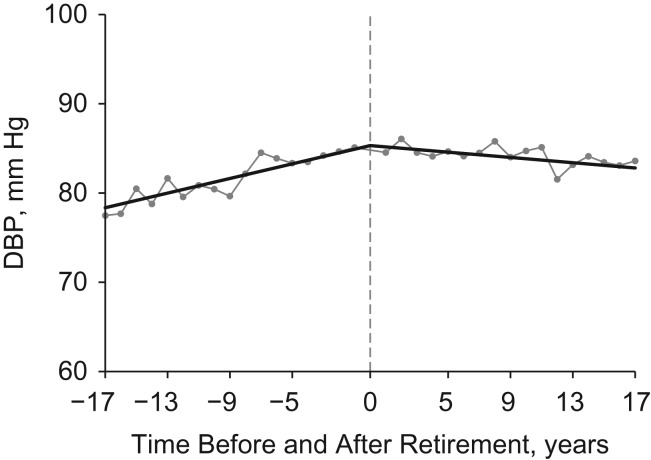
Trajectories of diastolic blood pressure (DBP) before and after retirement in the China Health and Nutrition Survey, 1991–2011. The straight lines are the predicted trajectories, separated at the year of retirement. The connected dots are the predicted mean values for DBP in each year. The time of retirement is year 0.

The addition of quadratic terms did not improve the model fits for SBP and DBP, so results from linear regression analyses are shown in Table 2. Positive preretirement slopes suggested that SBP and DBP increased with years before retirement, and negative postretirement slope changes suggested that the increasing preretirement slopes were reduced after retirement. The straight lines in Figures 1 and 2 show the trajectories of adjusted mean values for SBP and DBP. SBP increased over time both before and after retirement, but its growth rate was reduced after retirement. DBP increased over time before retirement, but decreased after retirement. The connected dots show the predicted mean values for blood pressure in each year. Dots are evenly distributed around the piecewise lines, suggesting that the piecewise models fitted the data well.

**Table 2.** Results From Piecewise Regression Analyses of the Association Between Retirement and Blood Pressure in the China Health and Nutrition Survey, 1991–2011

Slope	Systolic Blood Pressure (*n* = 1,084)			Diastolic Blood Pressure (*n* = 1,084)		
	β^a^	95% CI	*P* Value^b^	β^a^	95% CI	*P* Value^b^
Preretirement linear slope	0.791	0.657, 0.925	<0.001	0.410	0.325, 0.494	<0.001
Postretirement change in linear slope	−0.463	−0.663, −0.264	<0.001	−0.557	−0.682, −0.433	<0.001

Abbreviation: CI, confidence interval.

^a^ The model included adjustment for sex, province, age at retirement, age in 1991, highest educational qualification, preretirement characteristics (spouse's employment status (or no spouse), household income, occupational skill level, workplace physical activity level, smoking status, alcohol consumption, and body mass index), time-varying community urbanization index, and use of antihypertensive medication.

^b^*P* value for piecewise linear regression; tests of statistical significance were 2-sided.

**Figure 3.**
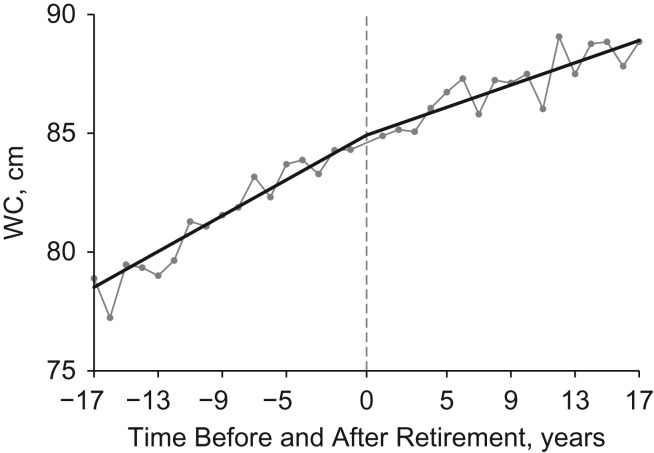
Trajectories of waist circumference (WC) before and after retirement in the China Health and Nutrition Survey, 1991–2011. The straight lines are the predicted trajectories, separated at the year of retirement. The connected dots are the predicted mean values of WC in each year. The time of retirement is year 0.

Table 3 shows results for adiposity measures. BMI changed with years before retirement in a quadratic shape (*P* < 0.01). The joint *P* value (assuming both linear and quadratic postretirement slope changes were zero) was 0.84, suggesting that the shape of the BMI curve did not change after retirement (the trajectory is shown in Web Figure 1, available at http://aje.oxfordjournals.org/). Adding quadratic terms did not improve the model fit for WC (*P* = 0.25). WC increased linearly with years before retirement, and the increasing preretirement slope was reduced after retirement (*P* < 0.01). Its trajectory (adjusted mean values) is shown in Figure 3.

**Table 3.** Results From Piecewise Regression Analyses of the Association Between Retirement and Adiposity Measures in the China Health and Nutrition Survey, 1991–2011

Time Period and Slope	Body Mass Index^a^ (*n* = 1,084)			Waist Circumference (*n* = 970)		
	β^b^	95% CI	*P* Value^c^	β^d^	95% CI	*P* Value^c^
Preretirement						
Linear slope	0.194	0.124, 0.264	<0.001	0.377	0.305, 0.448	<0.001
Quadratic slope	−0.004	−0.007, −0.001	<0.01			
Postretirement change^e^						
Linear slope change	0.011	−0.055, 0.077	0.75	−0.142	−0.249, −0.035	<0.01
Quadratic slope change	0.001	−0.002, 0.004	0.64			

Abbreviation: CI, confidence interval.

^a^ Weight (kg)/height (m)^2^.

^b^ Results were fully adjusted (see Table 2, footnote “a”) except for use of antihypertensive medication and preretirement body mass index.

^c^*P* value for piecewise linear regression; tests of statistical significance were 2-sided.

^d^ Results were fully adjusted (see Table 2, footnote “a”) except for use of antihypertensive medication.

^e^ Joint significance of the linear and quadratic slopes: *P* = 0.84.

**Figure 4.**
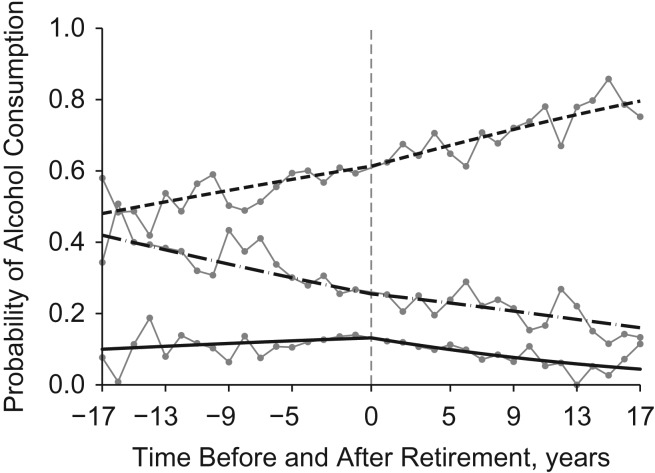
Trajectories of the probabilities of alcohol consumption before and after retirement in the China Health and Nutrition Survey, 1991–2011. The dashed line shows the probability of being a nondrinker. The dashed-and-dotted line shows the probability of being a light/moderate drinker (total amount of pure alcohol consumed: <60 g/day for men, <40 g/day for women). The solid line shows the probability of being a heavy drinker (total amount of pure alcohol consumed: ≥60 g/day for men, ≥40 g/day for women). The connected dots show the probabilities for each alcohol consumption status in each year. The time of retirement is year 0.

Multinomial odds ratios (i.e., exponential coefficients) from logistic regression analyses are shown in Table 4. The odds ratio for being a light/moderate drinker versus a nondrinker was not changed significantly after retirement, but the odds ratio for being a heavy drinker versus a nondrinker changed by 0.919 every year after retirement. In other words, the ratio of the probability of being a heavy drinker to the probability of being a nondrinker decreased by 8.1% (1 − 0.919) every year after retirement, compared with the preretirement pattern. We transformed odds ratios into predicted probabilities for each drinking status by averaging across all values of the covariates (Figure 4). The probability of being a nondrinker increased from 48% at the beginning of the analysis period to 61.3% at the year of retirement and to 79.6% 17 years after retirement. The probability of being a light/moderate drinker decreased from 42% to 25.5% at the year of retirement, and it further decreased to 16.6% 17 years after retirement. The probability of being a heavy drinker increased from 10% at the beginning of the analysis period to 13.2% at retirement but decreased to 4.4% at the end of the analysis period. The average marginal effect of a 1-year increase after retirement on the probability of being a heavy drinker was −0.008 (*P* < 0.001), suggesting that retirement was associated with a reduced probability of being a heavy drinker.

**Table 4.** Results From Piecewise Regression Analyses of the Association Between Retirement and Categorical Behavioral Risk Factors in the China Health and Nutrition Survey, 1991–2011

Consumption Level^a^ and Slope	Alcohol Drinking (*n* = 970)			Smoking (*n* = 1,084)		
	OR^b^	95% CI	*P* Value^c^	OR^d^	95% CI	*P* Value^c^
Light/moderate use versus nonuse						
Preretirement	0.912	0.882, 0.942	<0.001	0.950	0.927, 0.974	<0.001
Postretirement change	1.005	0.957, 1.057	0.83	1.003	0.965, 1.042	0.89
Heavy use versus nonuse						
Preretirement	0.955	0.918, 0.995	0.03	0.960	0.935, 0.985	<0.01
Postretirement change	0.919	0.862, 0.982	0.01	0.963	0.923, 1.005	0.08

Abbreviations: CI, confidence interval; OR, odds ratio.

^a^ Light/moderate smoking was defined as <20 cigarettes/day; heavy smoking was defined as ≥20 cigarettes/day. Light/moderate drinking was defined as <60 g/day for men and <40 g/day for women; heavy drinking was defined as ≥60 g/day for men and ≥40 g/day for women.

^b^ Results were fully adjusted (see Table 2, footnote “a”) except for use of antihypertensive medication and preretirement alcohol consumption.

^c^*P* value for piecewise logistic regression; tests of statistical significance were 2-sided.

^d^ Results were fully adjusted (see Table 2, footnote “a”) except for use of antihypertensive medication and preretirement smoking status.

The odds ratio for being a smoker versus a nonsmoker was not significantly changed after retirement. Trajectories in the probability are shown in Web Figure 2.

### Moderators

We further tested whether the association between retirement and CVD risk factors was moderated by sex or urbanicity. Results for moderating factors are shown in Table 5. Positive interaction terms for blood pressure suggested that retirement-related blood pressure changes were weaker for women than for men (for SBP, *P* < 0.01; for DBP, *P* = 0.03) and weaker for rural dwellers than for urban dwellers (for SBP, *P* < 0.01; for DBP, *P* = 0.04). There were no significant sex differences or urban-rural differences in the association between retirement and WC or BMI. Moderating tests were not conducted for alcohol consumption and smoking status, since very few women and rural dwellers were drinkers or smokers.

**Table 5.** Moderation of the Associations Between Retirement and Selected Cardiovascular Disease Risk Factors by Sex and Urbanicity in the China Health and Nutrition Survey, 1991–2011

CVD Risk Factor	No. of Participants in Analysis	Postretirement Change × Female Sex				Postretirement Change × Rural Residence			
		β	95% CI	*P* Value^a^	Joint *P* Value^b^	β	95% CI	*P* Value^a^	Joint *P* Value^b^
Systolic blood pressure, mm Hg	1,084	0.300	0.090, 0.509	<0.01		0.385	0.118, 0.652	<0.01	
Diastolic blood pressure, mm Hg	1,084	0.145	0.014, 0.276	0.03		0.170	0.008, 0.332	0.04	
Waist circumference, cm	970	0.065	−0.041, 0.172	0.23		0.072	−0.058, 0.202	0.28	
Body mass index^c^	1,084	−0.007^d^	−0.074, 0.060	0.84	0.51	−0.032^d^	−0.120, 0.055	0.47	0.19
		−0.001^e^	−0.006, 0.004	0.68		0.005^e^	−0.002, 0.011	0.14	

Abbreviations: CI, confidence interval; CVD, cardiovascular disease.

^a^*P* value for piecewise linear regression; tests of statistical significance were 2-sided.

^b^ Joint significance of the linear interaction term (i.e., linear slope × moderators) and the quadratic interaction term (i.e., quadratic slope × moderators).

^c^ Weight (kg)/height (m)^2^.

^d^ Coefficient of the linear postretirement change × moderator.

^e^ Coefficient of the quadratic postretirement change × moderator.

### Sensitivity analysis

A total of 156 participants went back to work after retirement. We repeated the analyses in a study sample with these participants excluded, and this did not change the original results (results not shown).

## DISCUSSION

We found that retirement in China was accompanied by a lower DBP, a slowdown in the increase of both SBP and WC over time, and a reduction in the probability of being a heavy alcohol drinker. We did not find any association between retirement and BMI or smoking.

Our results suggest, then, that retirement has a beneficial association with blood pressure, central obesity, and alcohol consumption, at least in the Chinese context. Hypertension and central obesity are important risk factors for CVD (26–29). For example, a meta-analysis has shown that a 20-mm Hg increase in SBP or a 10-mm Hg increase in DBP is associated with more than a doubling in the CVD death rate among people aged 40–69 years, and an even stronger association in older people (27). Another study using participants from 52 countries found that WC was strongly related to myocardial infarction risk, even after adjustment for BMI and height (29). Additionally, there is evidence that heavy drinking is associated with increased risks of having CVD and dying from CVD (30, 31), and a 15-year prospective cohort study of 220,000 men aged 40–79 years from 45 areas in China showed that CVD mortality increased linearly with alcohol consumption (22).

China is characterized by extreme rural-urban disparity. Long-term residents of urban areas in China usually work in formal sectors of the economy and therefore have formal wages, retire at mandatory ages, and receive generous pensions. On the contrary, most rural residents are self-employed in agriculture-related activities, with low incomes and pensions, and are not restricted by mandatory retirement ages (32). This study found a stronger beneficial association between retirement and blood pressure for urban dwellers. One possible explanation is that postretirement financial stress in rural areas has offset the beneficial association with retirement.

In addition, we have shown that the beneficial association between retirement and blood pressure were stronger for men than for women. It's likely that women's family-related labor burden increases after retirement, and high levels of family obligation, such as caregiving, may be associated with increased CVD (33, 34), which might reduce the beneficial association between retirement and blood pressure for women. Besides, men are more likely to have unhealthy dietary and drinking habits from coping with job stress (35). Stronger beneficial associations with retirement for men may be linked to a healthier lifestyle after retirement, such as less drinking.

### Comparison with previous studies

There are no previous longitudinal studies of retirement and CVD risk factors in China with which to compare our results, but our findings differ from those of several recent longitudinal studies conducted in Western counties which did not find any beneficial association between retirement and metabolic risk factors. For example, several studies based on the Health and Retirement Study in the United States, in comparison with either working people or people at the preretirement level, found that retirement was associated with increased BMI (10), weight (13, 36), and illness conditions (2). In another study using data from the English Longitudinal Study of Ageing, Behncke (1) reported that retirement increased the risk of having higher BMI and hypertension.

There are 3 possible reasons for the inconsistencies between our findings and the Western results. First, exposure to adverse working environments, particularly work-related stress, has been linked with increased risks of hypertension (37) and CVD (38, 39). Thus, if a job is perceived as less satisfying or more stressful, retirement may instead lead to better cardiovascular health outcomes. The working environment in China is generally less favorable than that in developed countries, and therefore retirement in China may be beneficial for the health of older workers. Second, the notion of “filial piety” is more salient in Chinese culture. Adult children in China are highly motivated to care for and support their parents financially and emotionally (40, 41), and the proportion living with aging parents in China is higher than that in Western countries (42–44). These strong family ties may buffer the impact of potentially stressful events in retirement for Chinese retirees. Third, men and women in China retire much earlier than people in the West. Early retirement has been suggested to be linked to poorer postretirement health in some Western studies (45, 46), but it might have a different health impact when retiring at a younger age is the social norm.

### Strengths and limitations

To our knowledge, this study is the only longitudinal study in China to have examined the association between retirement and CVD risk factors. The study benefited from a 17-year observation period in which participants were evaluated both before and after retirement and in which blood pressure and adiposity outcomes were objectively measured. A multilevel framework was used to account for between- and within-cluster effects (47). Missing values for the covariates were multiply imputed, which allowed for the use of information from all cases. An advantage of this study was the longitudinal analysis of the same individual both before and after retirement, which largely reduces the likelihood of reverse causality. We examined the slope change before and after retirement rather than the values themselves. If someone were retired due to increased levels of CVD risk factors, it would increase the mean values of the trajectories but not the slopes.

The response rate in the CHNS is 80%–88% across all waves relative to the previous wave, and 60% of individuals who participated in 1989 remained in the study in 2011. Thus, potential bias related to loss to follow-up is a general limitation of this study, as people with CVD are more likely to drop out and people who die of CVD are lost to follow-up. Those who remained in the study after retirement might have been healthier than those who retired after dropout. Migrant workers who move from rural areas to urban areas are also lost to follow-up, but their numbers should have been very small because rural-to-urban migration usually happens in younger workers, and migration was rare at the time members of the study samples were young (48).

Another limitation is that the CHNS data were not weighted to take account of the sampling design, as the sampling frame was not available at baseline. Thus, these results may not be generalizable to the population from which the sample was selected.

In conclusion, our study suggests that retirement may be beneficial for blood pressure, central obesity, and alcohol consumption in China, and it emphasizes the difference by sex and urbanicity in this association. Understanding the health impacts of retirement is essential if changes to retirement policy are to be cost-effective to the economy and beneficial to the health of China's older population.

## Supplementary Material

Web Material
