# Supplementary material for: The Associations Between Retirement and Cardiovascular Disease Risk Factors in China: A 20-Year Prospective Study
Source: Am J Epidemiol. 2017 Apr 5;185(8):688–96. doi: 10.1093/aje/kww166 (PMC5394248; doi:10.1093/aje/kww166)
Supplement: Web Material [file kww166xuewebmaterialfinal.pdf]

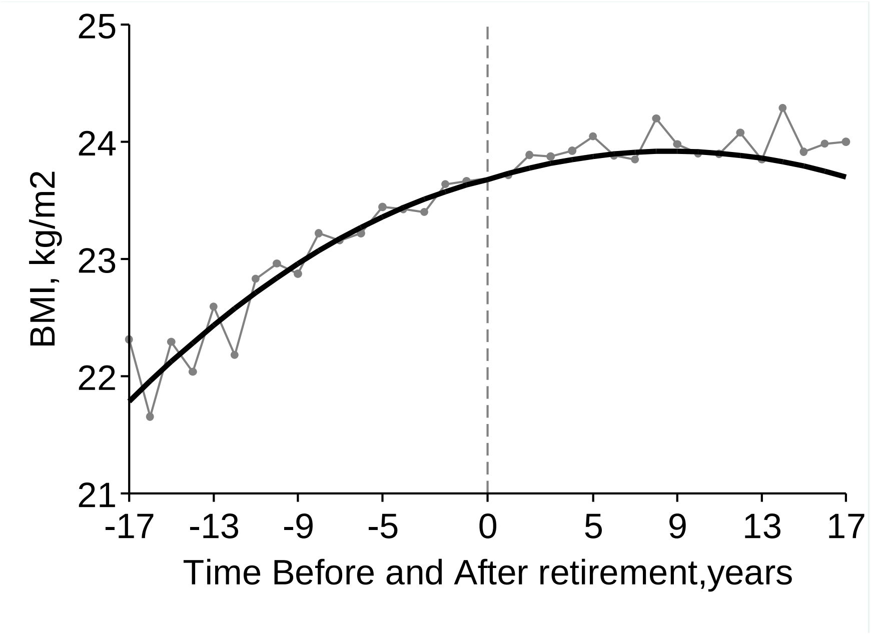

Web Figure 1. Trajectories of body mass index (BMI; weight (kg)/height (m)^2)) before and after retirement in the China Health and Nutrition Survey, 1991–2011. The solid curves show the predicted trajectories, separated at the year of retirement. The connected dots show the predicted mean values for BMI in each year. The time of retirement is year 0.

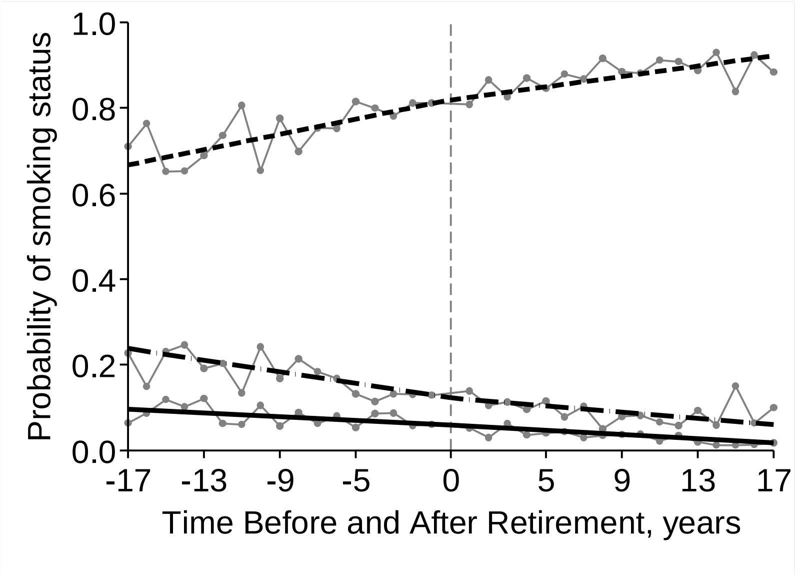

Web Figure 2. Trajectories of probabilities for each smoking status before and after retirement in the China Health and Nutrition Survey, 1991–2011. The dashed line shows the probability of being a nonsmoker. The dashed-and-dotted line shows the probability of being a light/moderate smoker. The solid line shows the probability of being a heavy smoker. The connected dots show the predicted probabilities for smoking status in each year. The time of retirement is year 0.
